# Metal migration and subunit swapping in ALS-linked SOD1: Zn^2+^ transfer between mutant and wild-type occurs faster than the rate of heterodimerization

**DOI:** 10.1016/j.jbc.2022.102610

**Published:** 2022-10-18

**Authors:** Chad M. Dashnaw, Ao Yun Zhang, Mayte Gonzalez, Jordan C. Koone, Bryan F. Shaw

**Affiliations:** Department of Chemistry and Biochemistry, Baylor University, Waco, Texas, USA

**Keywords:** motor neuron disease, copper, zinc, homeostasis, amyotrophic lateral sclerosis, heterodimerization, asparagine deamidation, ALS, amyotrophic lateral sclerosis, CE, capillary electrophoresis, DMF, dimethylformamide, ESI-MS, electrospray ionization mass spectrometry, SOD1, superoxide dismutase-1

## Abstract

The heterodimerization of WT Cu, Zn superoxide dismutase-1 (SOD1), and mutant SOD1 might be a critical step in the pathogenesis of SOD1-linked amyotrophic lateral sclerosis (ALS). Rates and free energies of heterodimerization (Δ*G*_Het_) between WT and ALS-mutant SOD1 in mismatched metalation states—where one subunit is metalated and the other is not—have been difficult to obtain. Consequently, the hypothesis that under-metalated SOD1 might trigger misfolding of metalated SOD1 by “stealing” metal ions remains untested. This study used capillary zone electrophoresis and mass spectrometry to track heterodimerization and metal transfer between WT SOD1, ALS-variant SOD1 (E100K, E100G, D90A), and triply deamidated SOD1 (modeled with N26D/N131D/N139D substitutions). We determined that rates of subunit exchange between apo dimers and metalated dimers—expressed as time to reach 30% heterodimer—ranged from t_30%_ = 67.75 ± 9.08 to 338.53 ± 26.95 min; free energies of heterodimerization ranged from Δ*G*_Het_ = -1.21 ± 0.31 to -3.06 ± 0.12 kJ/mol. Rates and Δ*G*_Het_ values of partially metalated heterodimers were more similar to those of fully metalated heterodimers than apo heterodimers, and largely independent of which subunit (mutant or WT) was metal-replete or metal-free. Mass spectrometry and capillary electrophoresis demonstrated that mutant or WT 4Zn-SOD1 could transfer up to two equivalents of Zn^2+^ to mutant or WT apo-SOD1 (at rates faster than the rate of heterodimerization). This result suggests that zinc-replete SOD1 can function as a chaperone to deliver Zn^2+^ to apo-SOD1, and that WT apo-SOD1 might increase the toxicity of mutant SOD1 by stealing its Zn^2+^.

Despite 3 decades of research, the basic mechanisms by which mutations in superoxide dismutase-1 (SOD1) trigger familial amyotrophic lateral sclerosis (ALS) remain a mystery (1). The autosomal nature of most (∼98%) of the ∼180 ALS-SOD1 mutations (2, 3, 4, 5) and the homodimeric nature of the SOD1 protein results in an early and ominous protein–protein interaction: heterodimerization of mutant and WT SOD1 (2, 6, 7, 8). Mutant/WT heterodimers are detected in ALS patients and cultured cells that coexpress WT and mutant SOD1 (9, 10, 11, 12).

Mutant/WT interactions (including heterodimerization) might explain the toxic synergy between WT SOD1 and ALS-mutant SOD1 in cultured cells and transgenic animals (2, 7, 12, 13, 14, 15). For example, the heterodimerization between WT SOD1 and ALS-variant SOD1 in HEK293 cells can promote toxicity independent of SOD1 aggregation (7). With A4V SOD1 transgenic mice, the coexpression of WT SOD1 appears to be necessary for pathogenesis (16, 17). In G93A SOD1 transgenic mice, the coexpression of human WT SOD1 increased the progression of symptoms nearly two-fold, compared to mice expressing only human G93A SOD1 (13, 18). Furthermore, the coexpression of human WT SOD1 and human G85R SOD1 in transgenic mice accelerates ALS onset two-fold, compared to mice expressing just human G85R SOD1 (13). Interestingly, the overexpression of human WT SOD1 along with *mouse* G85R SOD1 in transgenic mice reported no evidence of heterodimerization (or aggregation) as the mouse and human SOD1 proteins likely do not heterodimerize (14). These examples support the idea that the WT SOD1 protein is likely not—as long assumed—a benign or beneficial spectator in SOD1-linked familial ALS but somehow exacerbates (or in some cases triggers) the toxicity of mutant SOD1 (12, 19).

Two hypotheses can explain how WT SOD1 could increase mutant toxicity. The “template” hypothesis states that WT SOD1 directly binds to mutant SOD1 and triggers misfolding, for example, by increasing the half-life of mutant SOD1 or altering its structure (Fig. 1*A*). The “competition” hypothesis states that WT SOD1 outcompetes mutant SOD1 for protective factors, for example, chaperones or Cu^1+/2+^ or Zn^2+^ ions. Whether one SOD1 can steal metal ions from another SOD1 protein remains unknown (Fig. 1*B*) (20). Fully mature WT SOD1 protein is believed to be one of the tightest binders of intracellular Cu^1+^ (and Zn^2+^): K_d_ = 6.97 × 10^−21^ M for Cu^1+^; K_d_ = 8.46 × 10^−18^ M for Zn^2+^ (20, 21). Many ALS-variant SOD1 proteins exhibit mild-to-severely compromised affinity for both Cu^2+^ and Zn^2+^ (*i.e.*, the binding affinity for mature A4V and G93A are K_d_ = 1.68 × 10^−15^ M and K_d_ = 6.09 × 10^−17^ M respectively for Zn^2+^) (20, 22). This type of direct demetalation of mutant SOD1 by WT SOD1 would promote aggregation of the demetalated SOD1 by destabilization of the native state (23, 24).

**Figure 1:**
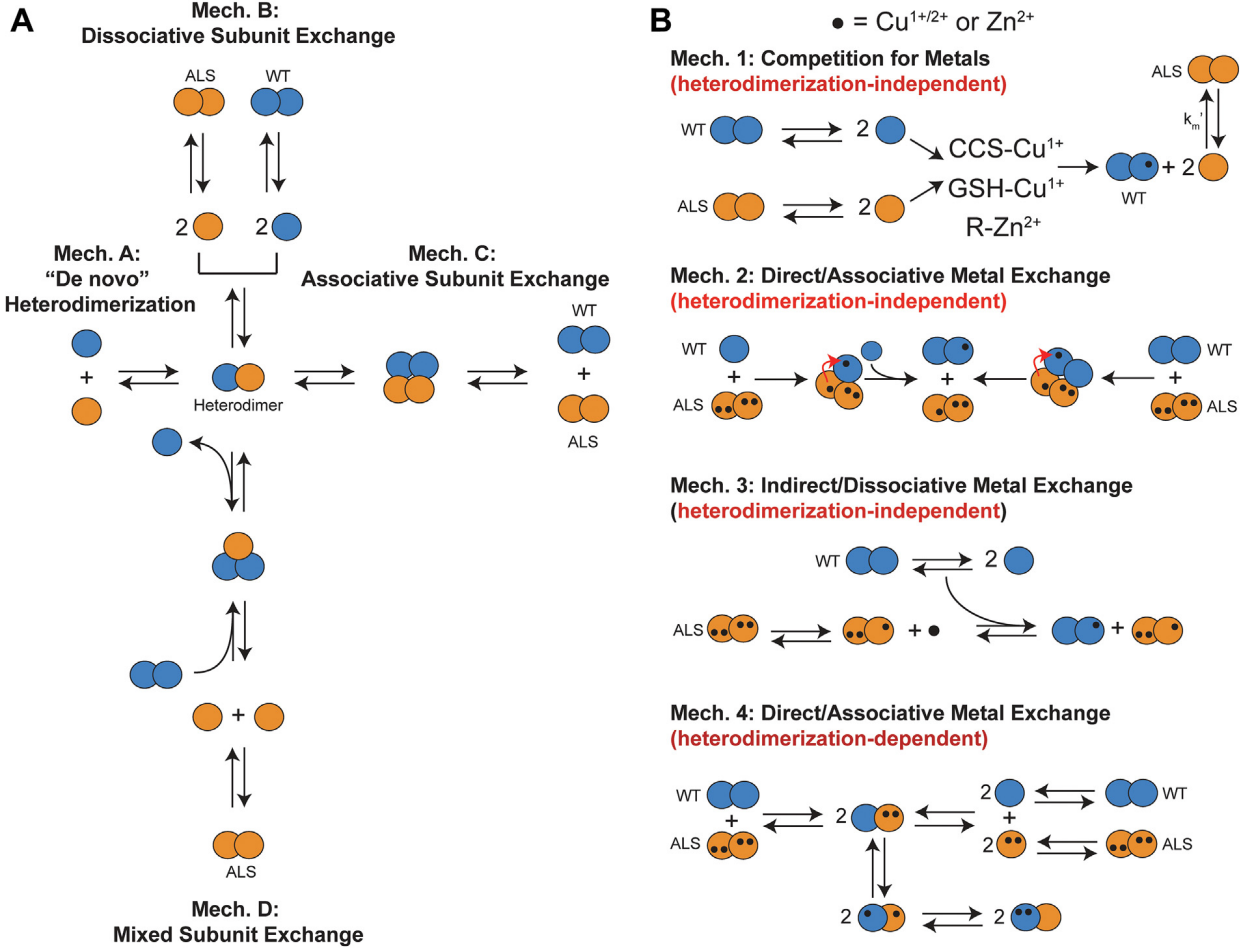
**Heterodimerization and metal transfer between ALS-variant SOD1 and WT SOD1.***A*, Possible mechanisms of heterodimerization between ALS-variant SOD1 and WT SOD1. *B*, Possible mechanisms of metal transfer between ALS-variant SOD1 and WT SOD1.

At the molecular level, little is known regarding SOD1 heterodimerization, including the mechanism(s) of heterodimerization (2, 15). Nearly all biophysical and biochemical studies of ALS-variant SOD1 have focused on homodimeric mutant SOD1 or WT SOD1 (25, 26, 27, 28, 29). Isolating heterodimers from equilibrium with both preceding homodimers is challenging. We have previously shown that the rate and free energy of heterodimerization between WT SOD1 and ALS-variant SOD1 can be measured using capillary electrophoresis (CE) (2). In a set of six biophysically diverse ALS-SOD1 variants, we found a correlation between patient survival time after diagnosis and Δ*G*_Het_ (R^2^ = 0.98 when G37R was excluded) (2). We found that rates of heterodimerization varied two-fold among mutant proteins in the metal free state and that Δ*G*_Het_ varied from −2.97 ± 0.13 to −1.15 ± 0.03 kJ mol^−1^ (2). Metal binding diminished the rate of subunit exchange by up to ∼38-fold (*i.e.*, from minutes to days) but only altered Δ*G*_Het_ by < 1 kJ mol^−1^ (2).

In theory, there are two types of protein heterodimerization: (i) *de novo* heterodimerization and (ii) subunit swapping (Fig. 1*A*). In *de novo* heterodimerization, monomeric WT and mutant SOD1 heterodimerize without prior homodimerization. Heterodimerization by subunit swapping of homodimeric WT or mutant SOD1 can involve three separate mechanisms: dissociative, associative, and mixed (Fig. 1*A*). Given SOD1’s long half-life in the central nervous system, subunit swapping undoubtedly occurs in the context of ALS (30).

## Results and discussion

Subunit swapping and metal migration between WT and mutant SOD1 homodimers is the subject of this study. The current paper investigated the heterodimerization between ALS-variant and WT SOD1 with imbalanced metalation states. Here, one homodimer is fully metalated and the other homodimer is metal-free; the resulting heterodimers are mixtures of each.

Detecting heterodimerization between WT and mutant homodimers can be analytically challenging when the two proteins differ by a single amino acid. With CE, heterodimerization can be easily observed so long as the heterodimer has a different electrophoretic mobility from the two preceding homodimers because of a difference in charge or drag. In these cases, CE can be used to quantify the rate of formation (k_Het_) of mutant-WT heterodimerization and equilibrium free energy of heterodimerization (Δ*G*_Het_). To our knowledge, CE is one of the only tools that can rapidly (in < 10 min) separate two forms of SOD1 that differ in mass by only ∼1 Da and differ in net charge (*Z*) by a single unit, while consuming nanoliters of solution.

We analyzed heterodimerization between WT SOD1 and four nonisoelectric mutants (E100K, E100G, D90A, and a triple mutant, N26D/N131D/N139D). The E100K, E100G, and D90A mutations are linked to ALS (2, 31). They occur at the surface of native SOD1, but in the β-sheet core of amyloid SOD1 (1). The triple N/D mutant is a model of WT SOD1 that has underwent asparagine deamidation (denoted “TD SOD1”) (32). This triply deamidated analog allows us to quantify rates and free energies of heterodimerization of pseudo-WT SOD1 with ALS mutants with poor resolution, such as E100G and D90A. Considering the long lifetime of SOD1 motor neurons, these deamidation products are also physiologically relevant (30). Deamidation at two of these three residues (N26 and N131) has been detected in SOD1 derived from human spinal cord (33). The heterodimerization between TD apo-SOD1 and A4V apo-SOD1 was also studied. However, the unstable A4V SOD1 protein produced a broad peak spanning ∼4 mobility units (possibly due to aggregation or binding to the capillary) which prevented the determination of ΔG_Het_ and rate of heterodimerization (Fig. S1).

For each capillary electrophoretic assay of heterodimerization, equal concentrations of the mutant SOD1 and WT SOD1 were mixed ([SOD1]_total_ = 30 μM dimer; [WT] = 15 μM dimer; [Mutant] = 15 μM dimer, per absorbance at 280 nm). This mixture was then analyzed with CE as a function of time. CE measurements were made until equilibrium was reached (between ∼200 min and ∼700 min depending upon metalation state). Four different heterodimerization experiments were conducted: (i) WT apo-SOD1 with mutant apo-SOD1, (ii) WT apo-SOD1 with mutant 4Zn-SOD1, (iii) WT 4Zn-SOD1 with mutant apo-SOD1, and (iv) WT 4Zn-SOD1 with mutant 4Zn-SOD1. The exponential decay function fit to the kinetic data for each ALS-mutant/WT heterodimer experiment was extrapolated to calculate the Δ*G*_Het_. At equilibrium (the plateau of each curve), Equation 1 was used to calculate K_Het_ which is then used to calculate Δ*G*_Het_ using Equation 2 (2).

(1) $$K_{Het}=\frac{{\left[WT-{ALS}_{Het}\right]}^{2}}{\left[{WT}_{\mathrm{Hom}}\right]\left[{ALS}_{\mathrm{Hom}}\right]}$$

(2) $$\Delta G_{Het}=-RT\;\ln\;K_{Het}$$

Each WT or mutant apo-SOD1 protein was remetalated with four equivalents of Zn^2+^ (per dimer) rather than with 2Cu^2+^ and 2Zn^2+^. Zinc titration was used to avoid mis-metalation (*i.e.*, Cu bound in Zn site or Zn bound in Cu site) (34). However, this zinc-replete state is also physiologically relevant (35, 36, 37). For example, SOD1 isolated from mice has been shown to contain a fraction of SOD1 subunits where Zn are bound at both the Zn and Cu binding sites, suggesting that 2Zn/subunit may be the preferred metalation state of SOD1 *in vivo*, prior to its interaction with the copper chaperone (35, 38). All experiments were performed under oxidizing conditions (with an intact intramolecular disulfide bond). Inductively coupled plasma mass spectrometry (ICP-MS) was used to quantify the amount of Zn in each sample following CE and mass spectrometry to ensure proper equivalents of Zn was present for each experiment. Less than 0.1 stoichiometric equivalents of Zn were present in dimeric apo-SOD1 and 3.7 to 3.9 equivalents of Zn were present in dimeric 4Zn-SOD1 (Table S1)

### Rates and free energies of heterodimerization between apo and zinc-replete homodimers

Examining the heterodimerization of the E100K SOD1 protein is especially suited for CE (as opposed to native mass spectrometry) because its mass is only ∼1 Da different than WT, but its net charge differs by 2 units/subunit.

**Figure 2:**
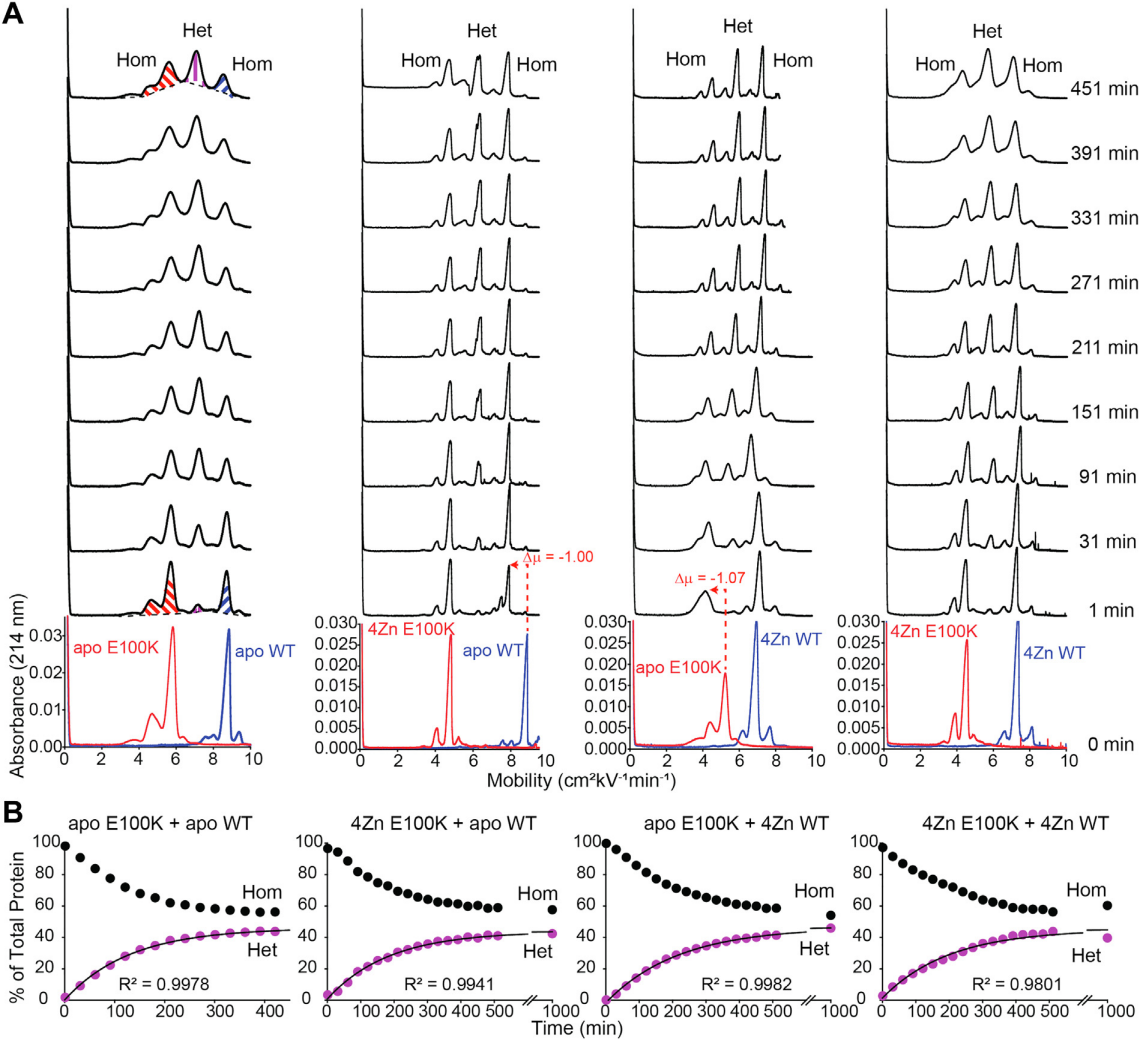
**Capillary electrophoresis of E100K and WT SOD1 heterodimerization.***A*, electropherograms before and after mixing homodimeric E100K SOD1 (*red*) and WT SOD1 (*dark blue*) at variable metalation states. Time of injection into the capillary is indicated to the right of the spectra. The homodimer (Hom) and heterodimer (Het) peaks are labeled in the t = 451 min electropherograms. *B*, kinetic plots of heterodimerization of E100K and WT SOD1 at variable metalation states. The break in the x-axis is from 600 to 900 min. Integration of electropherograms in (*A*) yields relative abundance of WT and ALS-variant SOD1 homodimers (Hom, *black*) and heterodimer (Het, *purple*), expressed as percent of total protein. CE performed at pH 7.4, 22 °C, storage at 15 °C; [SOD1]_total_ = 30 μM. ALS, amyotrophic lateral sclerosis; CE, capillary electrophoresis; SOD1, superoxide dismutase-1.

When both E100K and WT SOD1 are in the metal-free, disulfide-intact state, the rate constant of heterodimerization is k_Het_ = 0.76 ± 0.02 × 10^−2^ min^−1^. This rate is faster than any of the three different combinations of metalated E100K/WT SOD1; k_Het_ = 0.57 ± 0.03 × 10^−2^ (4Zn E100K/apo WT), k_Het_ = 0.47 ± 0.02 × 10^−2^ (apo E100K/4Zn WT), and k_Het_ = 0.47 ± 0.18 × 10^−2^ min^−1^ (4Zn E100K/4Zn WT) (Fig. 2, Table 1). We also expressed rates of heterodimerization as the time required for 30% of all protein to exist in the heterodimeric state, denoted t_30%_ (Table 1). Values ranged from t_30%_ = 137 to 336 min for the E100K/WT heterodimer mixed metalation studies.

**Table 1:** Thermodynamic and kinetic parameters of heterodimerization for WT and ALS-variant SOD1 with varied metalation states at 15 μM homodimer ([SOD1]_total_ = 30 μM), pH 7.4, 22 °C

ALS-variant and WT/TD SOD1 mixture	Rate constant (k_Het_; 10^−2^ min^−1^)	Rate (t_30%_; min)	Assumed K_Het_ (t = 5000 min)	Assumed Δ*G*_Het_ (t = 5000 min, kJ mol^−1^)
Apo E100K and apo WT	0.76 ± 0.02	136.91 ± 3.84	2.96 ± 0.18	−2.66 ± 0.07
4Zn E100K and apo WT	0.57 ± 0.03	206.42 ± 19.80	2.43 ± 0.31	−2.18 ± 0.14
Apo E100K and 4Zn WT	0.47 ± 0.02	217.25 ± 13.88	3.50 ± 0.17	−3.06 ± 0.12
4Zn E100K and 4Zn WT	0.47 ± 0.18	335.59 ± 2.30	1.94 ± 0.28	−1.63 ± 0.18
Apo E100G and apo TD	1.44 ± 0.19	67.75 ± 9.08	2.78 ± 0.35	−2.49 ± 0.32
4Zn E100G and apo TD	0.71 ± 0.11	221.39 ± 34.74	1.86 ± 0.50	−1.47 ± 0.62
Apo E100G and 4Zn TD	0.69 ± 0.10	179.07 ± 8.11	2.09 ± 0.31	−1.78 ± 0.40
4Zn E100G and 4Zn TD	0.74 ± 0.07	203.04 ± 54.00	1.64 ± 0.20	−1.21 ± 0.31
Apo D90A and apo TD	0.95 ± 0.14	98.23 ± 13.86	3.02 ± 0.34	−2.70 ± 0.28
4Zn D90A and apo TD	0.63 ± 0.03	181.06 ± 14.83	2.22 ± 0.18	−1.95 ± 0.20
Apo D90A and 4Zn TD	0.55 ± 0.04	213.27 ± 10.93	2.32 ± 0.12	−2.06 ± 0.12
4Zn D90A and 4Zn TD	0.72 ± 0.21	158.07 ± 60.84	2.48 ± 0.43	−2.21 ± 0.41
Apo TD and apo WT	1.28 ± 0.08	85.78 ± 5.79	2.34 ± 0.31	−2.08 ± 0.32
4Zn TD and apo WT	0.41 ± 0.04	296.53 ± 30.04	2.09 ± 0.14	−1.80 ± 0.17
Apo TD and 4Zn WT	0.66 ± 0.12	187.65 ± 37.79	2.27 ± 0.08	−2.01 ± 0.09
4Zn TD and 4Zn WT	0.38 ± 0.03	338.53 ± 26.95	2.13 ± 0.71	−1.79 ± 0.83

Electropherograms were analyzed using the skim method. Each experiment was run in triplicate.

The slowing of heterodimerization upon metal binding is expected as metalation of SOD1 stabilizes the homodimer (2). Since the rate constant of the other three combinations of metalated/apo states are comparable with each other, this suggests that only one homodimer needs to be fully metalated to slow the rate of heterodimerization. The rate of heterodimerization is not dependent upon which subunit (mutant or WT) is metalated (Table 1).

**Figure 3:**
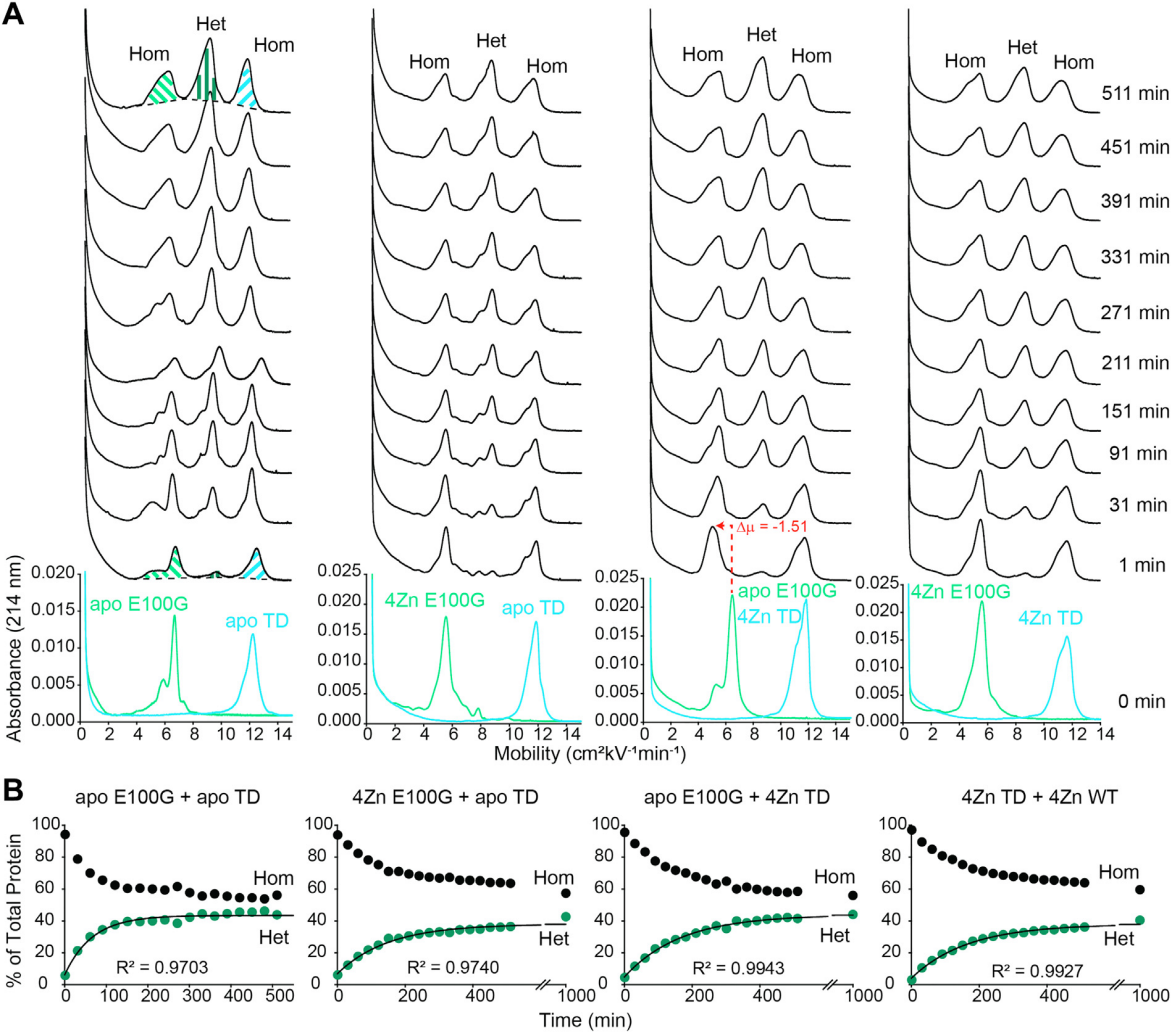
**Capillary electrophoresis of E100G and WT SOD1 heterodimerization.***A*, electropherograms before and after mixing of E100G SOD1 (*light green*) and triply deamidated (“TD”) SOD1 (*light blue*) of variable metalation states. Time of injection into the capillary is indicated to the right of the spectra. *B*, kinetic plot of heterodimerization for E100G and TD SOD1 of variable metalation states. The break in the x-axis is from 600 to 900 min. Integration of electropherograms in (*A*) yields relative abundance of WT and ALS-variant SOD1 homodimers (*black*) and heterodimer (*green*), expressed as percent of total protein. CE performed at pH 7.4, 22 °C, storage at 15 °C; [SOD1]_total_ = 30 μM. ALS, amyotrophic lateral sclerosis; CE, capillary electrophoresis; SOD1, superoxide dismutase-1.

**Figure 4:**
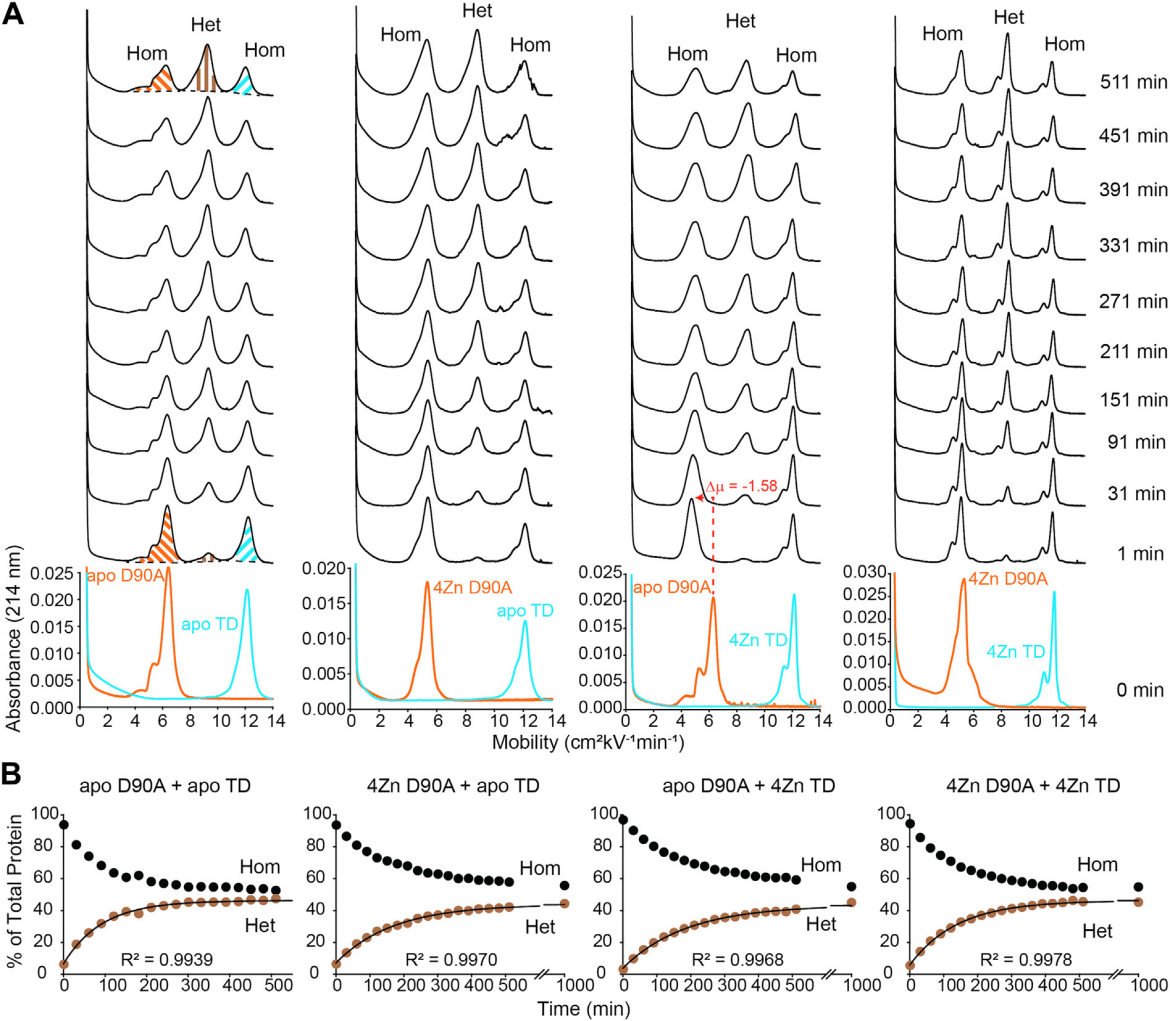
**Capillary electrophoresis of D90A and WT SOD1 heterodimerization.***A*, electropherograms before and after mixing of D90A SOD1 (*orange*) and TD SOD1 (*light blue*) of variable metalation states. *B*, kinetic plot of heterodimerization for D90A and TD SOD1 of variable metalation states. The break in the x-axis is from 600 to 900 min. Integration of electropherograms in (A) yields relative abundance of WT and ALS-variant SOD1 homodimers (*black*) and heterodimer (*brown*), expressed as percent of total protein. CE performed at pH 7.4, 22 °C, storage at 15 °C; [SOD1]_total_ = 30 μM. ALS, amyotrophic lateral sclerosis; CE, capillary electrophoresis; SOD1, superoxide dismutase-1.

Similar results were observed for both E100G and D90A when mixed with TD (Figs. 3 and 4, Table 1). The k_Het_ = 1.44 ± 0.19 × 10^−2^ min^−1^ for apo E100G/apo TD, compared to k_Het_ = 0.71 ± 0.11 × 10^−2^ (4Zn E100G/apo TD), k_Het_ = 0.69 ± 0.10 × 10^−2^ (apo E100G/4Zn TD), and k_Het_ = 0.74 ± 0.07 × 10^−2^ min^−1^ (4Zn E100G/4Zn TD) (Fig. 3, Table 1). When D90A and TD SOD1 are in the metal-free, disulfide-intact state, k_Het_ = 0.95 ± 0.14 × 10^−2^ min^−1^, compared to k_Het_ = 0.63 ± 0.03 × 10^−2^ (4Zn D90A/apo TD), k_Het_ = 0.55 ± 0.04 × 10^−2^ (apo D90A/4Zn TD), and k_Het_ = 0.72 ± 0.21 × 10^−2^ min^−1^(4Zn D90A/4Zn TD) (Fig. 4, Table 1). Although not as significant, these rate constants suggest that only one SOD1 subunit (mutant or WT) is required to be metalated for k_Het_ to decrease. This dominant effect of metalation is additional evidence in support of the associative mechanism of subunit exchange (Mech. C in Fig. 1) (2). Values for t_30%_ ranged from 68 to 221 min and 98 to 213 min for the E100G/TD and D90A/TD heterodimer mixed metalation studies respectively (Table 1).

**Figure 5:**
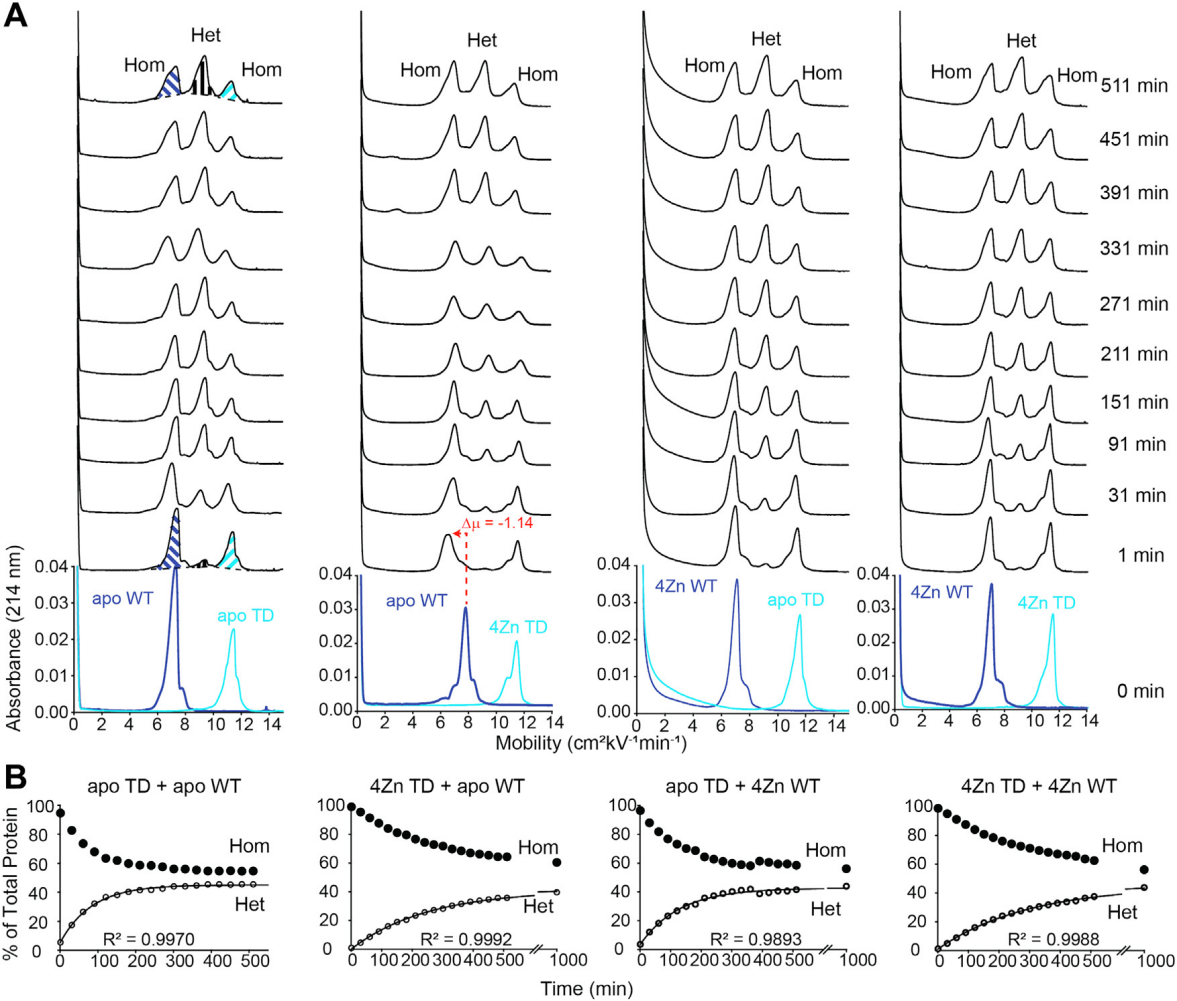
**Capillary electrophoresis of TD and WT SOD1 heterodimerization.***A*, electropherograms before and after mixing of TD SOD1 (*light blue*) and WT SOD1 (*dark blue*) of variable metalation states. *B*, kinetic plot of heterodimerization for TD and WT SOD1 of variable metalation states. The break in the x-axis is from 600 to 900 min. The relative abundance of WT and TD SOD1 homodimers (*black*) and heterodimer (*open*/*white*) was calculated by integrating the electropherograms above. CE performed at pH 7.4, 22 °C, storage at 15 °C; [SOD1]_total_ = 30 μM. CE, capillary electrophoresis; SOD1, superoxide dismutase-1.

The heterodimerization between WT and TD SOD1 was used as a WT/WT control of sorts (Fig. 5, Table 1). As expected, a slowing of the rate of heterodimerization was observed for TD and WT upon metalation. The k_Het_ = 1.28 ± 0.08 × 10^−2^ min^−1^ for apo TD/apo WT, compared to k_Het_ = 0.41 ± 0.04 × 10^−2^ (4Zn TD/apo WT), k_Het_ = 0.66 ± 0.12 × 10^−2^ (apo TD/4Zn WT), and k_Het_ = 0.38 ± 0.03 × 10^−2^ min^−1^ (4Zn TD/4Zn WT) (Fig. 5, Table 1). The t_30%_ values ranged from 86 to 339 min (Table 1).

**Figure 6:**
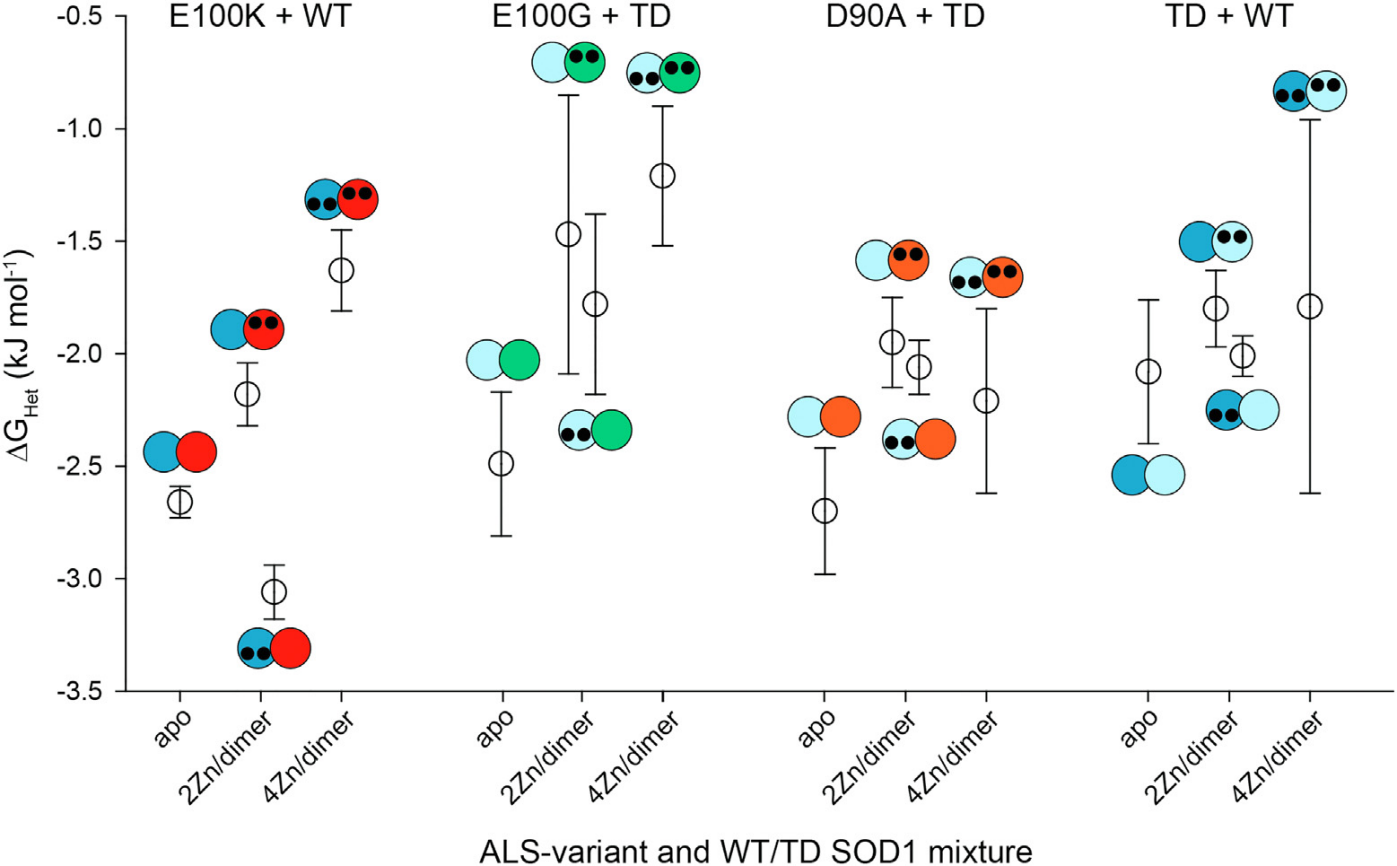
**Plot of the free energy of heterodimerization between severalALS-variantsand WT/TD SOD1 at varied metalation states.** WT (*dark blue*), E100K (*red*), E100G (*green*), D90A (*orange*), triply deamidated (“TD”) SOD1 (*light blue*). Error bars represent the SD. ALS, amyotrophic lateral sclerosis; SOD1, superoxide dismutase-1.

The Δ*G*_Het_ of E100K and WT SOD1 became less favorable with increasing metalation (except for one case). For WT and E100K apo-SOD1, the Δ*G*_Het_ = −2.66 ± 0.07 kJ mol^−1^ compared to Δ*G*_Het_ = −2.18 ± 0.14 kJ mol^−1^ (apo WT/4Zn E100K) and Δ*G*_Het_ = −1.63 ± 0.18 kJ mol^−1^ (4Zn WT/4Zn E100K). The free energy of heterodimerization for 4Zn WT and apo E100K seemed to be an outlier to the observed trend (Δ*G*_Het_ = −3.06 ± 0.12 kJ mol^−1^) (Figs. 2 and 6 and Table 1). The difference in Δ*G*_Het_ for 4Zn WT/4Zn E100K and apo E100K/apo WT is ΔΔ*G*_Het_ = + 1.03 ± 0.19 kJ mol^−1^. This positive value suggests that homodimers are stabilized by metal binding. It is possible that the metalated subunit stabilizes the unmetalated subunit of the partially metalated heterodimer, promoting heterodimerization in a cooperative mechanism similar to the “1Zn effect.” (39, 40).

For both E100G and D90A, metalation had similar effects on the free energy of heterodimerization with TD when compared with E100K and WT. The Δ*G*_Het_ = −2.49 ± 0.32 kJ mol^−1^for apo E100G/apo TD, compared to Δ*G*_Het_ = −1.47 ± 0.62 kJ mol^−1^ for 4Zn E100G/apo TD, Δ*G*_Het_ = −1.78 ± 0.40 kJ mol^−1^ for apo E100G/4Zn TD, and Δ*G*_Het_ = −1.21 ± 0.31 kJ mol^−1^ for 4Zn E100G/4Zn TD (Fig. 3, Table 1). The Δ*G*_Het_ = −2.70 ± 0.28 kJ mol^−1^ for apo D90A/apo TD, compared to Δ*G*_Het_ = −1.95 ± 0.20 for 4Zn D90A/apo TD, Δ*G*_Het_ = −2.06 ± 0.12 for apo D90A/4Zn TD, and Δ*G*_Het_ = −2.21 ± 0.41 kJ mol^−1^ for 4Zn D90A/4Zn TD (Fig. 4, Table 1). Again, a similar trend is observed, as the homodimers are metalated, Δ*G*_Het_ becomes slightly less favorable (Fig. 6).

The Δ*G*_Het_ for TD and WT are as follows; Δ*G*_Het_ = −2.08 ± 0.32 for apo TD/apo WT, Δ*G*_Het_ = −1.80 ± 0.17 for 4Zn TD/apo WT, Δ*G*_Het_ = −2.01 ± 0.09 for apo TD/4Zn WT, and Δ*G*_Het_ = −1.79 ± 0.83 kJ mol^−1^ for 4Zn TD/4Zn WT (Fig. 5, Table 1). The Δ*G*_Het_ between TD and WT was not significantly affected by variations in metalation state.

For each mutant chosen in this study, the electropherogram of each apo-SOD1 protein consisted of a single dominant peak (with the exception of A4V). Small satellite peaks were also observed at higher and lower mobility (±∼1 cm^2^kV^−1^min^−1^) and remained throughout the time course of the heterodimer experiments. These minor peaks have been observed in our previous analysis of SOD1 by CE (2, 32, 41, 42). Because electrophoretic mobility is the quotient of net charge and drag (shape), the peaks at lower mobility could be (in theory) due to perturbations that lower the magnitude of net negative charge and/or increase drag. Metal binding or protonation (41) as well as misfolded/aggregated states would cause protein peaks to shift to lower mobility. We can rule out metalation because the relative intensity of the satellite peaks does not correlate with the average metal stoichiometry determined by ICP-MS (Table S1). In contrast, the minor peaks at a higher mobility represent proteins with higher magnitudes of net negative charge and/or lower drag. These higher mobility peaks have been shown (for SOD1) to arise from deprotonation, asparagine deamidation, or cysteine oxidation (32, 41, 43).

### Putative metal migration between 4Zn-SOD1 and apo-SOD1

One intriguing observation in this study is the downward shift in mobility of either WT or mutant apo-SOD1, when either apo-SOD1 is mixed with fully metalated WT or mutant SOD1 (4Zn-SOD1). This shift poses an intriguing caveat to the interpretation set forth above. In the first electropherogram collected after mixing *any* apo-SOD1 with 4Zn-SOD1 (*e.g.*, WT apo-SOD1 with E100K 4Zn-SOD1 or WT 4Zn-SOD1 with E100K apo-SOD1), there is a rapid decrease in mobility and broadening of the apo-SOD1 peak (either WT or mutant apo-SOD1; Figure 2, Figure 3, Figure 4, Figure 5).

This shift in mobility (Δμ) of apo-SOD1 upon mixing with homodimeric 4Zn-SOD1 is equivalent to the Δμ associated with binding a single Zn^2+^ to the apo-SOD1 dimer (41). This suggests that the apo-SOD1 is rapidly acquiring one to two Zn^2+^ per dimer from the fully metalated homodimer (either WT or mutant 4Zn-SOD1) (Figure 2, Figure 3, Figure 4, Figure 5). This putative metalation of apo-SOD1 occurs faster than the rate of heterodimerization (within 10 min after mixing) and is not dependent upon heterodimerization. We hypothesize that the apo-SOD1 homodimer is “stealing” labile Zn^2+^ ions that are weakly bound to the metalated homodimer and/or unbound Zn^2+^ ions.

**Figure 7:**
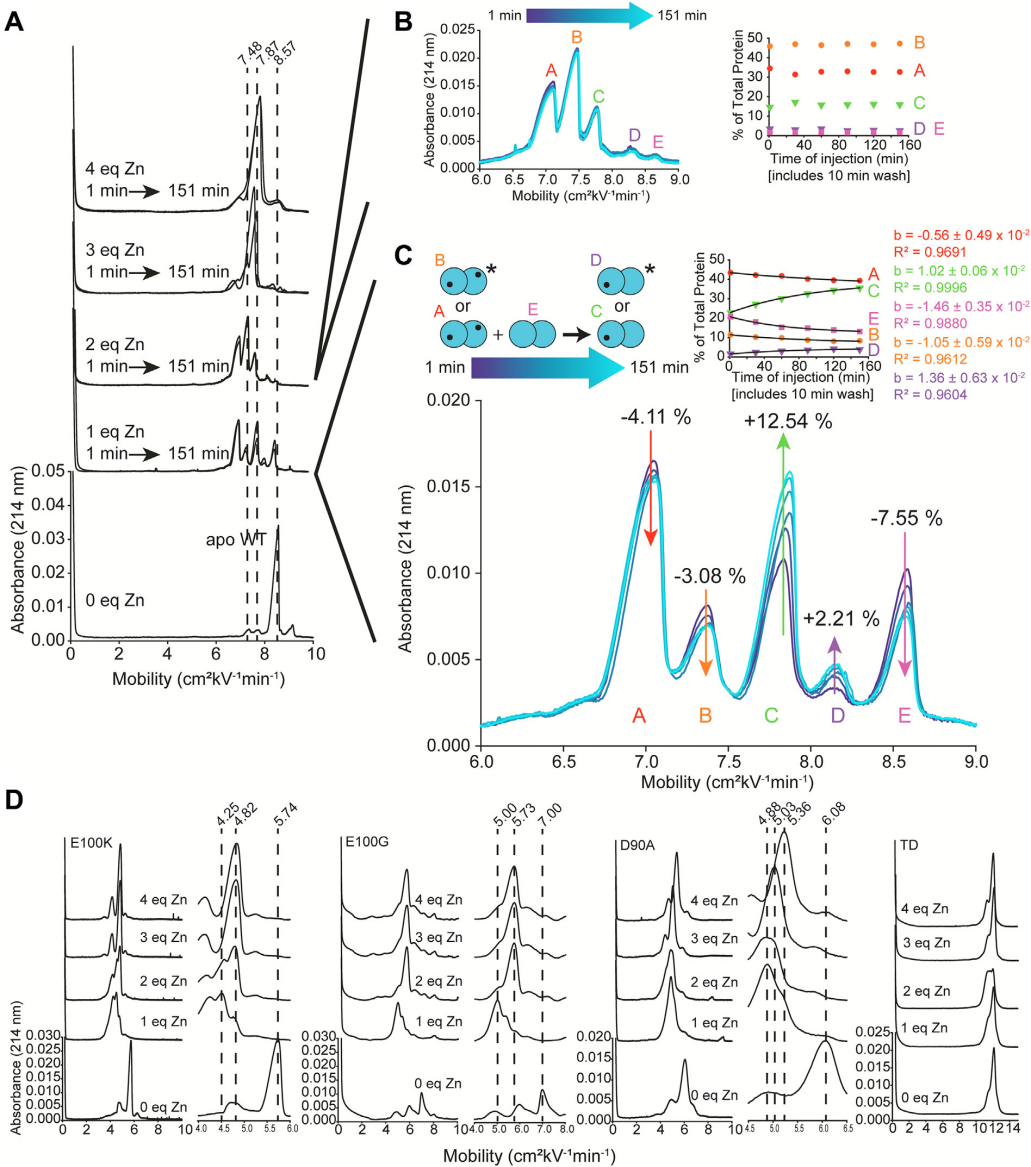
**Zn**^**2+**^**titration of ALS-variant and WT SOD1.***A*, capillary electropherograms of homodimeric WT before and after titration of Zn^2+^; pH 7.4, 22 °C; [SOD1] = 30 μM (stoichiometries listed per dimer). *B*, electropherograms and kinetic plots for the inorganic heterodimerization between WT 2Zn-SOD1 genetic homodimers. The relative abundance of WT inorganic homodimers and inorganic heterodimer was calculated by integrating electropherograms of 2Zn-SOD1 (“2 eq Zn”). Inset plot shows abundance of each peak in 2Zn-SOD1 sample. *C*, electropherograms and kinetic plots for the inorganic heterodimerization between WT 1Zn-SOD1 genetic homodimers. The relative abundance of WT inorganic homodimers and inorganic heterodimer was calculated by integrating the 1 eq Zn electropherograms. An exponential function (Equation 1) was fit to the plot of inorganic homodimer disappearance and inorganic heterodimer appearance. Inset plot shows abundance of each peak in 1Zn-SOD1 sample. *D*, capillary electropherograms of homodimeric mutant-SOD1 before and after titration of Zn; pH 7.4, 22 °C; [SOD1] = 30 μM with zoomed in regions highlighting Δμ for each metalation state. SOD1, superoxide dismutase-1.

A simultaneous increase in mobility or change in peak shape of the 4Zn-SOD1 protein would not be expected if this metalated SOD1 homodimer was losing < 2Zn/dimer as the electrophoretic mobility of 2Zn-SOD1, 3Zn-SOD1, and 4Zn-SOD1 are practically identical due to charge regulation (Fig. 7) (41). Thus, the transfer of a single metal ion from 4Zn-SOD1 to apo-SOD1 would only shift the mobility of apo-SOD1 upon binding Zn^2+^. ICP-MS confirmed that the Zn:SOD1 dimer ratio is approximately 4, prior to mixing with apo-SOD1 (*i.e.*, E100K = 3.81, WT = 3.72) (Table S1). It is possible that the “fourth” Zn^2+^ is weakly bound as it is bound to the Cu site (previous studies have shown that the binding of the first two Zn^2+^ ions are more thermodynamically favorable than the binding of the last two Zn^2+^ ions) (39).

Regarding this electrophoretic signature of zinc binding—and the interpretation of electrophoretic shifts—it must be remembered that the binding of Zn^2+^ to SOD1 affects the electrophoretic mobility of SOD1 in two offsetting ways (41). First, the binding of each Zn^2+^ imparts two formal units of positive charge to the protein complex (these changes can be compensated by “charge regulation,” *i.e.*, the changing of pK_a_ of ionizable residues in response to the binding of Zn^2+^). Second, Zn^2+^ binding decreases the effective hydrodynamic drag of the protein by ordering disordered loops (the first and second equivalents more so than the third and fourth) (41). These two effects—changes in net charge and drag—have been shown to vary per the exact zinc stoichiometry (41). In order to quantify effects of Zn^2+^ binding on mobility/peak shape under the current experimental conditions, we titrated Zn^2+^ into WT or mutant apo-SOD1 proteins (Fig. 7, Table 2). These data confirm that the downward shift in mobility upon the binding of two Zn^2+^ to homodimeric WT apo-SOD1 (Δμ = −1.09) is similar to the shift observed for WT apo-SOD1 when mixed with E100K 4Zn-SOD1 (Δμ = −1.00) (Figs. 2 and 7 and Table 2) or TD 4Zn-SOD1 (Δμ = −1.14) (Figs. 5 and 7 and Table 2). Similarly, the binding of two Zn^2+^ to homodimeric E100K apo-SOD1 (Δμ = −0.94) is similar to the shift observed for E100K apo-SOD1 when mixed with WT 4Zn-SOD1 (Δμ = −1.07) (Figs. 2 and 7 and Table 2).

**Table 2:** Mobility of WT, E100K, E100G, D90A, and TD SOD1 at varied metalation states

SOD1	μ of 0 Zn	μ of 1 Zn (Δμ)	μ of 2 Zn (Δμ)	μ of 3 Zn (Δμ)	μ of 4 Zn (Δμ)
WT	8.57	7.87 (−0.70)	7.48 (−1.09)	7.73 (−0.84)	7.98 (−0.59)
E100K	5.74	4.25 (−1.49)	4.80 (−0.94)	4.82 (−0.92)	4.83 (−0.91)
E100G	7.00	5.00 (−2.00)	5.73 (−1.27)	5.73 (−1.27)	5.73 (−1.27)
D90A	6.08	4.88 (−1.20)	4.85 (−1.23)	5.03 (−1.05)	5.36 (−0.72)
TD	12.13	12.12 (−0.01)	11.47 (−0.66)	12.13 (0.00)	12.14 (+0.01)

The downward shift in mobility upon the binding of two Zn^2+^ to homodimeric E100G apo-SOD1 (Δμ = −1.27) is similar to the shift observed for E100G apo-SOD1 when mixed with TD 4Zn-SOD1 (Δμ = −1.51) (Figs. 3 and 7 and Table 2). Similarly, the downward shift in mobility upon the binding of two Zn^2+^ to homodimeric D90A apo-SOD1 (Δμ = −1.58) is similar to the shift observed for D90A apo-SOD1 when mixed with TD 4Zn-SOD1 (Δμ = −1.23) (Figs. 4 and 7 and Table 2). We did not observe a downward shift in mobility for TD apo-SOD1 upon Zn binding, but rather peak broadening (Figure 3, Figure 4, Figure 5 and 7 and Table 2). These data suggest that 4Zn-SOD1 (mutant or WT) is prone to losing ∼ 2Zn^2+^ to apo-SOD1 (mutant or WT) upon mixing. If 4Zn-SOD1 lost more than 2Zn^2+^, a change in mobility/shape would be expected for the metalated protein peak in the electropherogram which was not observed (Figure 2, Figure 3, Figure 4, Figure 5).

### Mass spectral evidence of metal migration

Metal transfer between WT SOD1 and ALS-mutant SOD1 (or vice versa) has not been previously reported or studied to our knowledge. The binding of Zn^2+^ ions to proteins can be analytically challenging to detect because Zn^2+^ is undetectable by optical or magnetic spectroscopy. However, CE and mass spectroscopy can detect Zn^2+^ binding. Although the D90A and E100G mutations result in a large change in mass (ΔMW = −44.02 and −72.06 Da respectively), the E100K mutation has a ΔMW = −0.94 MW (nearly indistinguishable from WT SOD1) and is not suitable for the electrospray ionization mass spectrometry (ESI-MS) studies.

**Figure 8:**
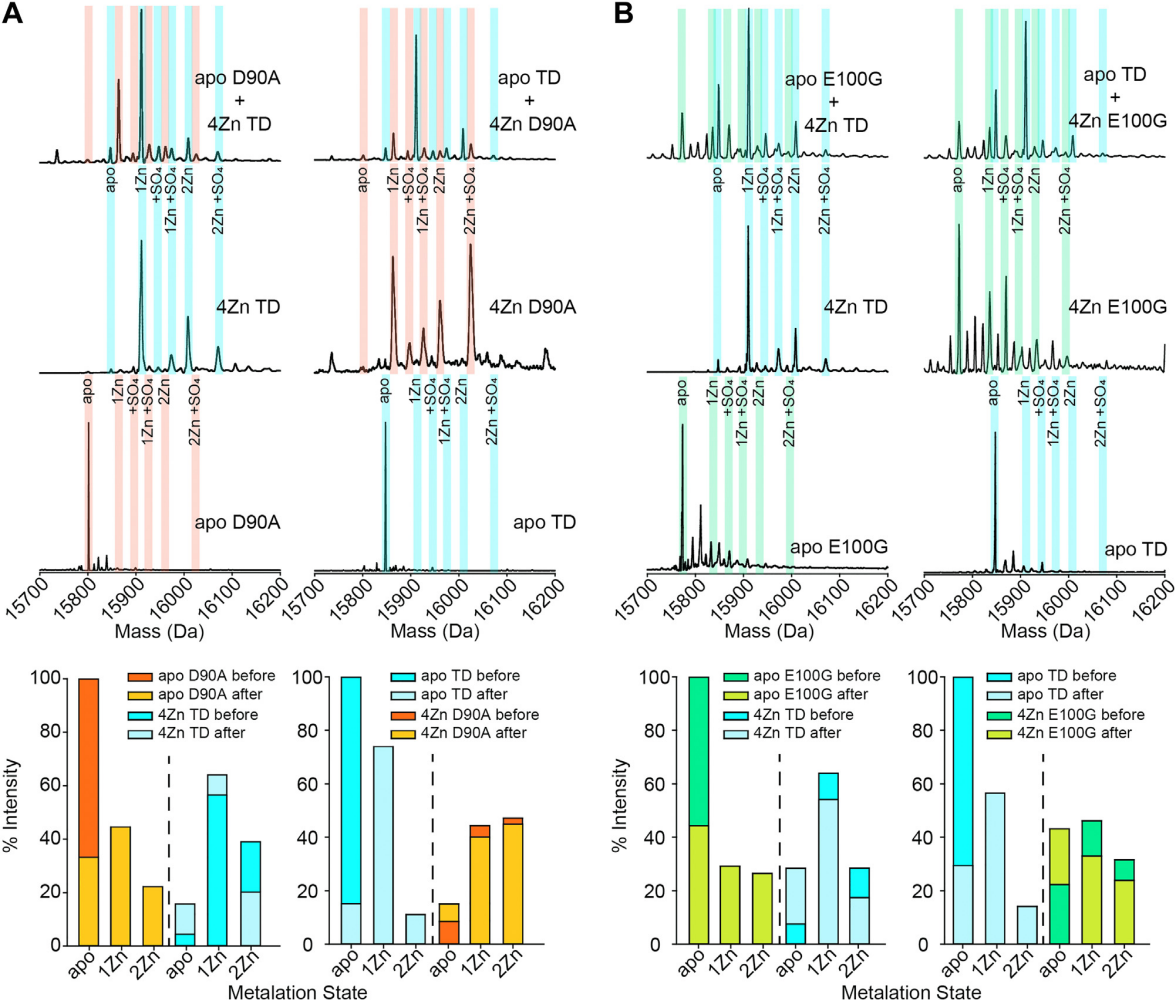
**Metal migration observed in SOD1 by mass spectroscopy.***A*, mass spectra of D90A and TD apo-SOD1 before and after heterodimerization with TD or D90A 4Zn-SOD1, respectively (monomerization occurs during ionization, but metal binding is preserved). Each spectra were integrated to determine the percentage of each metalation state for each SOD1 monomer and plotted. *B*, spectra of monomeric E100G and TD apo-SOD1 before and after heterodimerization with TD or E100G 4Zn-SOD1, respectively. Each spectra were integrated to determine the percentage of each metalation state for each SOD1 monomer and plotted. Protein samples (50 μl, [SOD1]_total_ = 30 μM) were diluted 4-fold into 30% acetonitrile, 100 μM formic acid immediately prior to injection into the mass spectrometer. Parameters of ESI were as follows: capillary voltage, 2.5 kV; source temperature, 100 °C; sampling cone voltage, 40 V; source offset voltage, 80 V; cone gas 50 l/h; purge gas 100 l/h. Deconvolution of raw spectra was achieved through the MaxEnt1 module in MassLynx software. SOD1, superoxide dismutase-1.

We used semi-soft ionization conditions (30% acetonitrile, 100 mM formic acid) that dissociate the dimer, but preserve metal binding, thus allowing us to determine which subunit is binding metals (44). Protein samples were washed into MQ and metalated prior to heterodimerization. After metalation and after heterodimerization, each sample was incubated at 4 °C for 48 h before ESI-MS analysis. Both D90A and E100G (apo or 4Zn) were mixed with TD (4Zn or apo) (Fig. 8*A*). The apo SOD1 spectra all showed a single primary peak at their corresponding molecular weights; 15801, 15772, and 15847 Da for D90A, E100G, and TD, respectively. Thus, all mass spectra of the apo-SOD1 showed no evidence of metalated protein (Fig. 8*A*).

The mass spectra of the 4Zn-SOD1 had several peaks which correlated to the 1Zn or 2Zn/subunit species with or without a sulfate adduct (Zn^2+^ = +65 Da, SO_4_^2−^ = +96 Da). As a monomer, the maximum number of Zn^2+^ is 2 as there are only two metal-binding active sites per monomer (44) (additional weak sites presumably exist elsewhere on the surface of the protein) (36, 45, 46). Note that trace mass spectral signals of apo-SOD1 are observed in 4Zn-SOD1 samples (Fig. 8*A*). This is likely due to metal loss during ionization as CE showed no evidence of apo-SOD1 in 4Zn-SOD1 samples before heterodimerization.

For TD 4Zn-SOD1, integration of spectra yielded 5.99% apo, 60.28% 1Zn, and 33.74% 2Zn before heterodimerization with any protein. It is important to note that for ESI-MS analysis, ionization efficiency for apo, 1Zn, and 2Zn-SOD1 species were assumed to be the same which of course they are likely not. As such, integration of peak intensities is more qualitative than quantitative (Table S2).

After mixing TD 4Zn-SOD1 with D90A apo-SOD1, integration of the peaks for TD 4Zn-SOD1 yielded 15.70% apo, 64.09% 1Zn, and 20.21% 2Zn. Integration of the peaks for D90A apo-SOD1 yielded 33.23% apo, 44.59% 1Zn, and 22.18% 2Zn. This shows that TD 4Zn-SOD1 is losing Zn^2+^ ions and D90A apo-SOD1 is binding Zn^2+^ ions (Fig. 8*A*). It is unclear whether D90A apo-SOD1 is acquiring Zn^2+^ from TD 4Zn-SOD1 during the 48 h incubation or if the metalated TD is losing metal ions during ionization where the D90A apo-SOD1 then acquires Zn^2+^ during electrospray ionization. However, the shift in mobility of the D90A apo-SOD1 peak upon mixing D90A apo-SOD1 and TD 4Zn-SOD1 during CE is likely due to D90A acquiring Zn^2+^ which suggests ionization is not required for metal exchange (Fig. 4*A*).

For D90A 4Zn-SOD1, integration of the mass spectrum yielded 8.45% apo, 44.38% 1Zn, and 47.17% 2Zn before heterodimerization. After mixing with TD apo-SOD1, integration of the peaks for D90A 4Zn-SOD1 yielded 14.99% apo, 40.05% 1Zn, and 44.96% 2Zn. Integration of the peaks for TD produced 15.08% apo, 73.90% 1Zn, and 11.02% 2Zn (Fig. 8*A*). Interestingly, only small differences are observed between spectra obtained when mixing D90A apo-SOD1 with TD 4Zn-SOD1 or when mixing D90A 4Zn-SOD1 with TD apo-SOD1. In both circumstances, mass spectrometry suggested either apo-SOD1 protein (D90A or TD) acquires Zn^2+^ ions while either metalated SOD1 simultaneously loses Zn^2+^ ions (Fig. 8*A*).

For E100G 4Zn-SOD1, integration of the mass spectrum yielded 22.28% apo, 46.20% 1Zn, and 31.52% 2Zn before heterodimerization. After mixing with TD apo-SOD1, integration of the peaks for E100G yielded 43.18% apo, 32.95% 1Zn, and 23.87% 2Zn. Integration of the peaks for TD corresponded to 29.35% apo, 56.52% 1Zn, and 14.13% 2Zn (Fig. 8*B*). As previously described, only small differences are observed between spectra obtained when mixing E100G apo-SOD1 with TD 4Zn-SOD1 or when mixing E100G 4Zn-SOD1 with TD apo-SOD1. Again, for both experiments, apo-SOD1 acquires Zn^2+^ ions and the metalated SOD1 loses Zn^2+^ ions (Fig. 8*B*). These mass spectra correlate with the CE electropherograms, which suggest that 4Zn-SOD1 (mutant or WT) is prone to losing ∼2 Zn^2+^ ions to apo-SOD1 (mutant or WT) upon mixing (Figure 2, Figure 3, Figure 4, Figure 5 and 7).

**Figure 9:**
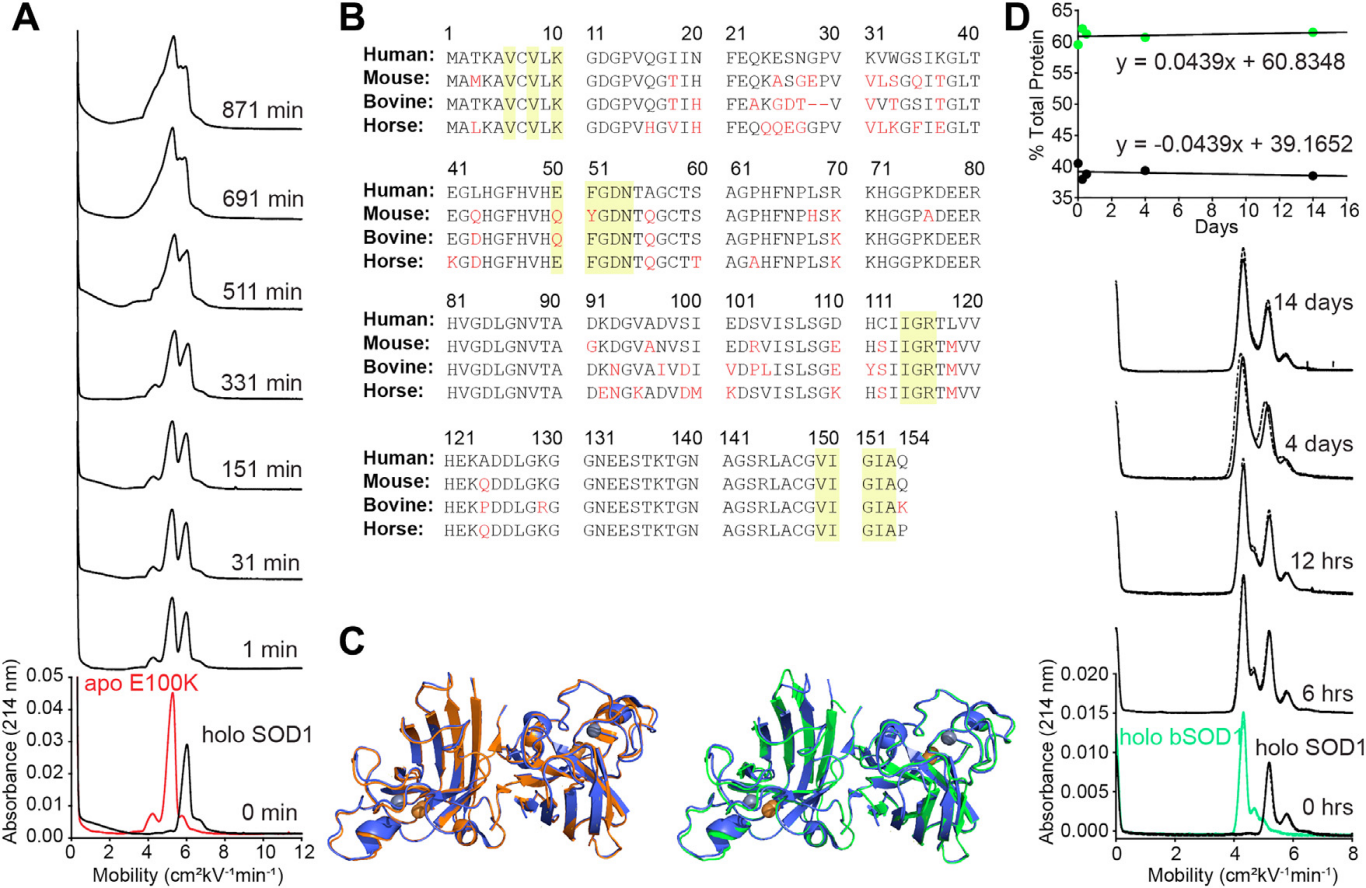
**Assessing heterodimerization between human holo-SOD1 and E100K apo-SOD1 or bovine holo-SOD1.***A*, electropherograms before and after mixing of E100K apo-SOD1 (*red*) and holo-SOD1 from human blood (*black*). Time of injection into the capillary is indicated to the right of the spectra. Concentration of protein was [SOD1]_holoSOD1_ = 15 μM; [SOD1]_E100K_ = 15 μM dimer, for a total concentration of [SOD1]_total_ = 30 μM; CE done at 22 °C, storage at 15 °C; pH 7.4. *B*, homology of human, mouse, bovine, and horse SOD1 protein sequence. Amino acids highlighted in *yellow* are at the dimer interface. Amino acids shown in *red* highlight changes in protein sequence compared to human SOD1. *C*, the PyMOL structure of human SOD1 (*blue*, PDB: 2C9V) is shown superimposed onto mouse SOD1 (*orange*, PDB: 3GTT) and bovine SOD1 (*green*, PDB: 1CBJ). *D*, electropherograms before and after mixing of holo-bovine SOD1 (holo bSOD1, *green*) and holo-SOD1 from human blood (*black*). Time of injection into the capillary is indicated to the right of the spectra. Concentration of protein was [SOD1]_holoSOD1_ = 15 μM; [SOD1]_bSOD1_ = 15 μM dimer, for a total concentration of [SOD1]_total_ = 30 μM; CE done at 22 °C, storage at 15 °C; pH 7.4. Integration of homodimer peaks was done over time. CE, capillary electrophoresis; SOD1, superoxide dismutase-1.

If one or two Zn^2+^ ions are migrating from one subunit to another, these Zn^2+^ ions are likely lost from the copper-binding site (where they might be bound more weakly) and migrating to the zinc site of the apo subunit (47). To quickly explore this possibility, properly metalated human WT 2Cu, 2Zn-SOD1 (isolated from human erythrocytes, purchased from Sigma Aldrich; holo-SOD1) was mixed with E100K apo-SOD1 (Fig. 9*A*). Holo-SOD1 and E100K apo-SOD1 are not well resolved during CE and their heterodimerization is illustrated by a merging of the two peaks (Fig. 9*A*).

The poor resolution between the holo-SOD1 and E100K apo-SOD1 homodimer peaks prevents the accurate determination of the rate and ΔG_Het_ between E100K apo-SOD1 and holo-SOD1. However, this set of electropherograms is still useful because it demonstrated that the E100K apo-SOD1 did not undergo a downward shift in mobility upon mixing with properly metalated WT holo-SOD1 (as it did with WT 4Zn-SOD1, Fig. 2*A*). Although WT holo-SOD1 still heterodimerizes with E100K apo-SOD1 (apparent from the merging of the two homodimer peaks with a central heterodimer peak), holo-SOD1 does not transfer metals to E100K apo-SOD1 according to CE. This suggests—but does not prove—that the Zn^2+^ ion(s) acquired by apo-SOD1 (mutant or WT) is transferred from the copper site and not the zinc site (47).

Since Zn^2+^ is spectroscopically silent (excluding x-ray radiation), one major limitation of this study is that we do not know which binding sites are involved with Zn^2+^ transfer. Given the long timescale of the heterodimerization measurements, it is assumed that the binding sites with the strongest affinity for Zn^2+^ are involved. We assume that the 4Zn-SOD1 derivative involves two Zn^2+^ ions bound at the Cu/Zn active sites (as previous titrations with Co^2+^ suggests binding at the active site per UV-vis) (41). We cannot rule out, however, the transfer of Zn^2+^ ions to weaker binding sites outside of the active site (36, 45, 46).

The exchange of metal ions between ALS-variant and WT SOD1 can occur through three possible mechanisms: direct exchange, indirect exchange, or inter-subunit exchange (Fig. 1). In the direct exchange mechanism, WT and ALS-variant SOD1 collide (as either monomeric or dimeric SOD1), and metal ions will transfer from one SOD1 to the other. In the indirect exchange mechanism, metal ions dissociate from SOD1 and reassociate with a different, less metalated SOD1. In the inter-subunit exchange mechanism, the heterodimer between WT and ALS-variant SOD1 is formed first, and then metal transfer occurs between the subunits. These three mechanisms are not mutually exclusive. The rapid rate that metals transfer from 4Zn-SOD1 to apo-SOD1 (Figure 2, Figure 3, Figure 4, Figure 5) should rule out heterodimerization-dependent metal exchange (Mech. 4 in Fig. 1).

### Inorganic heterodimers of genetic homodimers: one WT 2Zn-dimer and one WT apo-dimer yield two WT 1Zn-dimers

When using CE to interpret metalated SOD1 species or metal transfer between proteins, it must be remembered that a homodimeric SOD1 protein with one bound Zn^2+^ per dimer (*i.e.*, 1Zn-SOD1) should exhibit its own dynamic electropherogram as a result of heterodimerization (Fig. 7) (47). That is, two 1Zn/dimers could undergo subunit swapping to produce a dimer with two coordinated Zn^2+^ and one dimer that is metal free. To further investigate this type of inorganic heterodimer, we titrated 1Zn^2+^ into either WT or mutant apo-SOD1 homodimers (Fig. 7). After titrating 1Zn into an apo-SOD1 homodimer, we immediately injected the solution into the CE and collected longitudinal sets of electropherograms out to ∼150 min postmixing (Fig. 7).

Note the peak shape and splitting of WT 1Zn-SOD1 into three predominant peaks (Fig. 7*C*). The peak splitting is dynamic, on the timescale of heterodimerization, suggesting that these species are dimeric and undergo subunit exchange with each other (Fig. 7*C*). This triplet has been previously observed for WT 1Zn-SOD1 (at a single time point) under slightly different conditions (41). We assign the prominent peak on the far right (Fig. 7*C*) as apo-SOD1, the middle prominent peak as 1Zn-SOD1 (1Zn per dimer), and the far left peak as 2Zn-SOD1 (1Zn per subunit). Interestingly, at first, the major species seems to be 2Zn-SOD1 (1Zn per subunit) and as equilibrium is reached the population of 1Zn-SOD1 increases. This is indicative of subunit swapping between 2Zn-SOD1 (1Zn per subunit) and apo-SOD1 (Fig. 7*C*). This also may indicate that WT SOD1 prefers to form inorganic heterodimers as opposed to remaining an apo homodimer. The minor peaks might be caused by mis-metalation (34). For example, if a zinc ion binds the zinc site rather than the copper site, it would likely have a larger impact on the three-dimensional structure, due to stabilization of the zinc binding loops, and thus the protein would migrate slightly faster (Fig. 7*C*). Integration of the peaks rules out 2Zn-SOD1 with both Zn^2+^ ions being in the same subunit because this would exceed the amount of added Zn^2+^ in solution. Note that the triplet splitting of mutant 1Zn-SOD1 (Fig. 7*D*) is not as resolved as the splitting in the WT 1Zn-SOD1 proteins studied (Fig. 7*C*). After the addition of 2 equivalents of Zn^2+^, the same dynamic equilibrium was not observed but rather a static distribution of peaks was observed (Fig. 7*B*).

### “Minotaur” SOD1—Chimeric SOD1 heterodimers are unlikely, but why?

Since this is a paper about heterodimerization, we wondered whether SOD1 proteins from different organisms might heterodimerize. Knowing whether this type of heterodimerization actually occurs is important for interpreting experiments where human WT SOD1 is coexpressed in a nonhuman system expressing its own endogenous SOD1 (*e.g.*, human WT SOD1 coexpressed with mouse G86R SOD1 in a transgenic mouse (13)). We examined the sequence homology of a few SOD1 proteins (Fig. 9*B*). The amino acid sequence of mouse SOD1 is quite homologous with human SOD1 (83.8%); cow is lower (81.8%); horse SOD1 is even lower than cow (80.5%). For these three organisms, the sequence homology of the residues in the dimer interface is higher: 89.2, 97.4, 100% for mouse, cow, and horse, respectively (Fig. 9*C*) (48, 49).

We mixed properly metalated bovine 2Cu, 2Zn-SOD1 (bSOD1) that was isolated from cow erythrocytes with human 2Cu, 2Zn-SOD1 (hSOD1) isolated from human erythrocytes. These two proteins differ in their formal net charge by approximately 2 units (hSOD1 *Z*_CE_ = −14.26/dimer, bSOD1 *Z*_CE_ = −12.10/dimer) (50) and have similar mass. Both proteins were purchased from Sigma-Aldrich and used without further purification. These two proteins are known to be properly metalated, fully, with two equivalents of copper and zinc in each subunit. Each protein also possesses a small satellite peak during CE, due to the deamidation of a single asparagine residue (N26) (50). Surprisingly, after 14 days, no heterodimer peak emerged, suggesting that bSOD1 and hSOD1 do not heterodimerize with each other (Fig. 9*D*). The integration of both homodimer peaks remained constant over time (Fig. 9*D*). Considering the high sequence homology of the dimer interface (97.4%), the lack of heterodimerization suggests that residues outside of the actual dimer interface are critical to SOD1’s ability to undergo subunit swapping. We find this result to be a fascinating example of the complexities of molecular recognition.

### Conclusion: the physiological implications of heterodimerization of mixed metalation states and metal migration

The ALS-variants studied in this paper (E100K, E100G, and D90A) all demonstrated that metalation (partial or complete) of SOD1 slowed heterodimerization with WT or deamidated WT SOD1, making heterodimerization less favorable than that of mutant apo-SOD1 and WT or deamidated WT apo-SOD1 (Figure 2, Figure 3, Figure 4, Figure 5, Figure 6). Although the Δ*G*_Het_ increases upon metalation, heterodimerization was always energetically favorable (negative), which suggests that the partially metalated heterodimer is more stable than the apo homodimer. These heterodimerization studies of mixed metalation states supports the previously reported hypothesis that zinc-deficient SOD1 is stabilized by metalated SOD1, which may increase its half-life and toxicity (40). For example, Estévez *et al.* reported that Cu, Zn-SOD increases zinc-deficient SOD toxicity on nontransgenic motor neurons by stabilizing the “toxic intermediate” of zinc-deficient SOD by heterodimerization (40). Furthermore, it was shown that the enhanced toxicity was independent of whether the zinc-deficient SOD was WT or ALS mutant (40).

Moreover, all ALS-variants studied in this paper demonstrated mutual migration of Zn^2+^ between WT SOD1 (*i.e.*, apo-SOD1 acquired approximately ∼2 Zn^2+^ from 4Zn-SOD1) (Figure 2, Figure 3, Figure 4, Figure 5 and 8). “Metal migration” between WT and mutant subunits could explain how the presence of WT SOD1 renders some mutant SOD1 proteins more toxic. WT SOD1 could possibly outcompete or remove metal ions from ALS-variant SOD1 proteins with compromised affinity for both Cu^2+^ and Zn^2+^ (*i.e.*, A4V and G93A) (20, 21, 22). In this scenario, metal migration might be nonmutual and exclusive from the ALS-variant to WT.

Copper and zinc protect SOD1 from aggregation by promoting disulfide formation and stabilizing the native state (51). SOD1 selfassembles into a prion/amyloid-like species when disulfide-reduced and after losing (or never acquiring) its metal ions. Regardless of the mechanism by which apo-SOD1 is formed, the demetalation of mutant SOD1 promotes misfolding/aggregation (51, 52). Therefore, it is possible that WT SOD1 increases the toxicity of mutant SOD1 by diminishing the metal content of the mutant SOD1. If heterodimerization is required—in some cases—for “metal snatching,” the favorable free energy of SOD1 heterodimerization (Δ*G*_*Het*_) might explain the short patient survival time (after diagnosis) of biophysically benign (“cryptic”) mutations such as D101N (2).

## Experimental procedures

### Human SOD1 purification and characterization

Recombinant SOD1 (WT and ALS-variants) were expressed and purified using previously published protocols (2, 41, 42). Briefly, Yep351-hSOD1 plasmids were transfected into EG118Δsod1 yeast. Primary cultures of yeast stocks were grown to A_600 nm_ ∼1.5 (approximately 36 h) in YPD media and transferred to larger secondary cultures for growth for ∼7 days. Cells were then lysed, and SOD1 was purified through ammonium sulfate precipitation followed by three successive chromatographic separations: (i) hydrophobic interaction chromatography; (ii) size-exclusion chromatography; and (iii) ion-exchange chromatography. Solutions of SOD1 were characterized via gel electrophoresis and mass spectrometry. Protein concentrations were determined via UV-vis spectroscopy; ε_280 nm_ = 10,800 cm^−1^M^−1^ for apo-SOD1.

### Demetalation and remetalation of SOD1

Solutions of SOD1 were demetalated via dialysis over 6 days (2 days in each buffer solution, changed every 8 h): (i) 100 mM ammonium acetate, 5 mM EDTA, pH 3.8; (ii) 100 mM ammonium acetate, 100 mM NaCl, pH 3.8; and (iii) 100 mM ammonium acetate, pH 5.5. Metal content of apo-SOD1 was verified via ICP-MS to be less than 0.1 equivalents of copper and zinc per dimeric SOD1. All glassware was rinsed with solutions of 10 mM EDTA, followed by ultra-pure Milli-Q water to prevent metal contamination. Remetalation with zinc was accomplished by titrating four equivalents of ZnSO_4_ into the protein sample prior to CE analysis. Metal content of the metalated SOD1 solutions (and of the buffer) were confirmed via ICP-MS. Solutions of SOD1 were considered zinc replete (*i.e.*, 4Zn-SOD1), when the protein solution contained > 3.7 equivalents of zinc per dimeric SOD1, and the buffer contained < 0.05 equivalents of zinc per dimeric SOD1 in the protein solution.

### Inductively coupled plasma mass spectrometry

The extent of metalation was measured via a 7900 Agilent ICP-MS following CE and mass spectrometry heterodimer experiments. Protein solutions were classified as “metal free” (<0.1 equivalents of metal were present per dimeric SOD1) or “zinc replete” (>3.7 equivalents of zinc per dimeric SOD1). All buffer solutions contained < 0.1 equivalents of Zn per dimeric SOD1. All unmixed protein solutions showed a single dominant peak during electrophoresis, indicating the presence of only a single species (*i.e.*, ALS-mutant 4Zn-SOD1 or WT apo-SOD1), and ancillary peaks likely represent different protonation states, misfolded states, deamidation, or cysteine oxidation, which have been previously observed (2, 32, 41, 43).

### Capillary electrophoresis

CE was performed at 29 kV in a bare fused-silica capillary on a Beckman P/ACE instrument. Incubation of WT and mutant SOD1 occurred at 15 to 22 °C, that is, solutions were incubated at 15 °C in the CE sample rack (chilled), but brief electrophoresis occurs at 22 °C. The capillary was kept at 22 °C by a liquid cooled outer jacket to prevent Joule heating. The time was recorded at the time the sample was placed inside the instrument and not at the time of injection of the sample into the capillary. Before sample injection, the capillary was reconditioned with 0.1 M NaOH, Milli-Q water, and 10 mM potassium phosphate, pH 7.4. Electroosmotic flow was measured by adding 1 μl of 100 mM dimethylformamide (DMF) to the protein solution prior to injection, for a final concentration of [DMF] = 2 mM.

### Thermodynamic analysis of electropherograms

Electropherograms were integrated using Origin software. The area corresponding to each dimer (homodimer or heterodimer) was then converted into percent total protein and plotted against time. Each plot was fit to Equation 3 to obtain a rate constant as well as t_30%_. Data was measured for 1022 min (36 replicates). For most of the metalated samples, 1022 min was not long enough to reach equilibrium. Using each fit, synthetic points were calculated at t = 5000 min which were then used to calculate the K_Het_ and ΔG_Het_ values. Repetitive CE led to baseline shifts/broadening of peaks which caused the integration of the homodimer peak closest to the DMF peak to be lower than expected. To avoid this artifact from affecting the K_Het_ and ΔG_Het_ values, the homodimer concentrations were assumed to be the same at t = 5000 min to obtain assumed K_Het_ and assumed ΔG_Het_ values.

(3) $$f=y_{0}+a\left(1-e^{\left(-bx\right)}\right)$$

### Electrospray ionization mass spectrometry

D90A, E100G, and TD SOD1 were washed into Milli-Q water (pH ∼ 5.5) and metalated with four equivalents of zinc sulfate. Each protein was incubated at 4 °C for 48 h before mixing to initiate heterodimerization. Heterodimers were incubated at 4 °C for 48 h before ESI-MS analysis. All solutions of SOD1 were analyzed for metal transfer via direct injection with a Waters Synapt G2 HDMS. Solutions were diluted to [SOD1]_total_ = 7.5 μM in 100 μM formic acid and 30% pure acetonitrile immediately prior to injection (*i.e.*, ∼1 min prior to detection). For heterodimerization studies, [SOD1]_TD_ = 3.75 μM, [SOD1]_Mutant_ = 3.75 μM. Parameters of ESI were as follows: capillary voltage, 2.5 kV; source temperature, 100 °C; sampling cone voltage, 40 V; source offset voltage, 80 V; cone gas 50 l/h; purge gas 100 l/h. Deconvolution of raw spectra was achieved through the MaxEnt1 module in MassLynx software.

## Data availability

All data are contained within the article.

## Supporting information

This article contains supporting information.

## Conflict of interest

The authors declare no conflict of interest.
