# Supplementary material for: Metal migration and subunit swapping in ALS-linked SOD1: Zn2+ transfer between mutant and wild-type occurs faster than the rate of heterodimerization
Source: J Biol Chem. 2022 Oct 18;298(12):102610. doi: 10.1016/j.jbc.2022.102610 (PMC9667317; doi:10.1016/j.jbc.2022.102610)
Supplement: Supplemental Figure and Tables [file mmc1.docx]

**Metal migration and subunit swapping in ALS-linked SOD1: Zn^2+^ transfer between mutant and wild-type occurs faster than the rate of heterodimerization**

Chad M. Dashnaw, Ao Yun Zhang, Mayte Gonzalez, Jordan C. Koone, and Bryan F. Shaw^*^

Department of Chemistry and Biochemistry, Baylor University, Waco, TX

^*^To whom correspondence should be addressed: [bryan_shaw@baylor.edu](mailto:bryan_shaw@baylor.edu)

**** Supporting Information ****

**
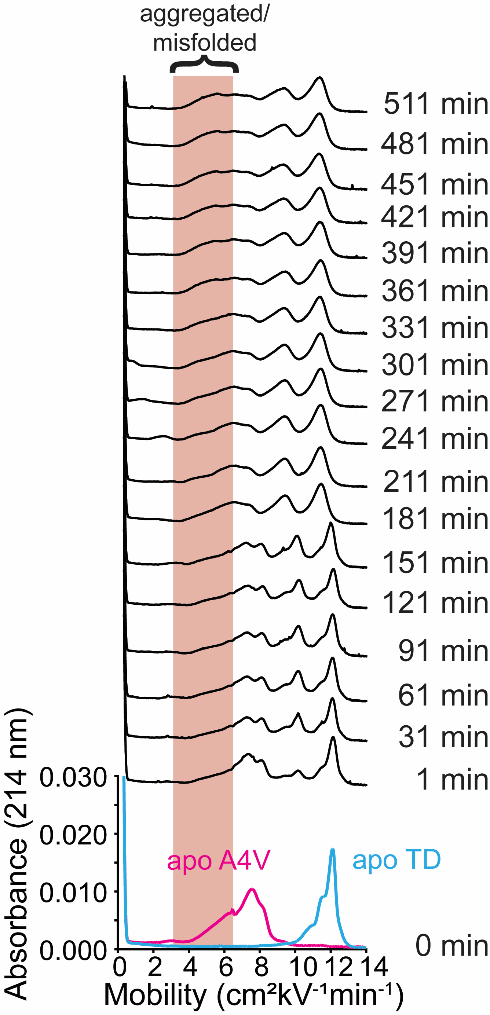
**

**Figure S1.** Electropherograms of A4V apo-SOD1 (pink) and TD apo-SOD1 (light blue) before and after mixing. Time of injection into the capillary is indicated to the right of the spectra. The orange shaded region indicates possible aggregated/misfolded states of A4V apo-SOD1. CE performed at pH 7.4, 22 °C, storage at 15 °C; [SOD1]_total_ = 30 μM.

**Table S1.** Metal equivalents of zinc per dimeric SOD1, as determined via ICP-MS. Protein solutions were classified as “metal free” (fewer than 0.1 equivalents of metal were present per dimer) or “zinc replete” (greater than 3.7 equivalents of zinc, but less than 4 total metal equivalents per dimer). Buffer used for capillary electrophoresis was also tested for zinc after each experiment.

|  | Zn:apo SOD1 dimer | Zn:metalated SOD1 dimer | Buffer |
| --- | --- | --- | --- |
| WT | 0.01 | 3.72 | 0.10 |
| E100K | -0.06 | 3.81 | -0.20 |
| E100G | 0.06 | 3.88 | 0.06 |
| D90A | -0.08 | 3.78 | -0.21 |
| TD | -0.16 | 3.85 | -0.16 |

**Table S2.** Raw integration of mass spectra for SOD1 samples before and after (shaded) heterodimerization.

|  | Apo | Apo + SO_2_^+^ | 1Zn | 1Zn + SO_2_^+^ | 2Zn | 2Zn+SO_2_^+^ |
| --- | --- | --- | --- | --- | --- | --- |
| Apo D90A | 7.78E+08 |  |  |  |  |  |
| Apo D90A |  | 5.36E+06 | 5.32E+06 | 1.88E+06 | 2.29E+06 | 1.29E+06 |
| 4Zn TD | 1.09E+06 | 3.71E+06 | 4.99E+07 | 1.18E+07 | 2.58E+07 | 1.68E+07 |
| 4Zn TD | 9.34E+05 | 1.64E+06 | 8.35E+06 | 2.16E+06 | 1.71E+06 | 1.61E+06 |
|  |  |  |  |  |  |  |
| 4Zn D90A |  | 1.75E+07 | 5.23E+07 | 3.97E+07 | 2.71E+07 | 7.06E+07 |
| 4Zn D90A | 4.61E+06 | 6.91E+06 | 1.93E+07 | 1.15E+07 | 1.56E+07 | 1.89E+07 |
| Apo TD | 7.98E+08 |  |  |  |  |  |
| Apo TD | 7.19E+06 | 9.17E+06 | 6.16E+07 | 1.85E+07 | 1.19E+07 |  |
|  |  |  |  |  |  |  |
| Apo E100G | 2.14E+08 | 3.70E+07 |  |  |  |  |
| Apo E100G | 2.03E+08 | 1.62E+08 | 1.19E+08 | 1.21E+08 | 1.38E+08 | 7.97E+07 |
| 4Zn TD | 6.36E+06 | 8.03E+06 | 8.87E+07 | 3.30E+07 | 3.42E+07 | 1.99E+07 |
| 4Zn TD | 2.37E+08 | 1.18E+08 | 5.09E+08 | 1.68E+08 | 1.61E+08 | 5.71E+07 |
|  |  |  |  |  |  |  |
| 4Zn E100G | 2.53E+08 | 1.64E+08 | 2.14E+08 | 1.26E+08 | 1.24E+08 | 1.08E+08 |
| 4Zn E100G | 5.97E+07 | 5.13E+07 | 4.71E+07 | 3.76E+07 | 3.71E+07 | 2.43E+07 |
| Apo TD | 1.14E+08 | 2.84E+07 |  |  |  |  |
| Apo TD | 8.75E+07 | 3.62E+07 | 1.90E+08 | 4.77E+07 | 4.62E+07 | 1.33E+07 |
